# Hepatitis B Screening and Vaccination Strategies for Newly Arrived Adult Canadian Immigrants and Refugees: A Cost-Effectiveness Analysis 

**DOI:** 10.1371/journal.pone.0078548

**Published:** 2013-10-18

**Authors:** Carmine Rossi, Kevin Schwartzman, Olivia Oxlade, Marina B. Klein, Chris Greenaway

**Affiliations:** 1 Department of Epidemiology, Biostatistics, and Occupational Health, McGill University, Montreal, Canada; 2 Centre for Clinical Epidemiology and Community Studies, Jewish General Hospital, Montreal, Canada; 3 Respiratory Epidemiology and Clinical Research Unit, Montreal Chest Institute, Montreal, Canada; 4 Respiratory Division, McGill University Health Centre, Montreal, Canada; 5 Department of Epidemiology, Harvard School of Public Health, Boston, Massachusetts, United States of America; 6 Department of Medicine, Divisions of Infectious Diseases/Immunodeficiency, McGill University Health Centre, Montreal, Canada; 7 Division of Infectious Diseases, Jewish General Hospital, Montreal, Canada; Copenhagen University Hospital Gentofte, Denmark

## Abstract

**Background:**

Immigrants have increased mortality from hepatocellular carcinoma as compared to the host populations, primarily due to undetected chronic hepatitis B virus (HBV) infection. Despite this, there are no systematic programs in most immigrant-receiving countries to screen for chronic HBV infection and immigrants are not routinely offered HBV vaccination outside of the universal childhood vaccination program.

**Methods and findings:**

A cost-effective analysis was performed to compare four HBV screening and vaccination strategies with no intervention in a hypothetical cohort of newly-arriving adult Canadian immigrants. The strategies considered were a) universal vaccination, b) screening for prior immunity and vaccination, c) chronic HBV screening and treatment, and d) combined screening for chronic HBV and prior immunity, treatment and vaccination. The analysis was performed from a societal perspective, using a Markov model. Seroprevalence estimates, annual transition probabilities, health-care costs (in Canadian dollars), and utilities were obtained from the published literature. Acute HBV infection, mortality from chronic HBV, quality-adjusted life years (QALYs), and costs were modeled over the lifetime of the cohort of immigrants. Costs and QALYs were discounted at a rate of 3% per year. Screening for chronic HBV infection, and offering treatment if indicated, was found to be the most cost-effective intervention and was estimated to cost $40,880 per additional QALY gained, relative to no intervention. This strategy was most cost-effective for immigrants < 55 years of age and would cost < $50,000 per additional QALY gained for immigrants from areas where HBV seroprevalence is ≥ 3%. Strategies that included HBV vaccination were either prohibitively expensive or dominated by the chronic HBV screening strategy.

**Conclusions:**

Screening for chronic HBV infection from regions where most Canadian immigrants originate, except for Latin America and the Middle East, was found to be reasonably cost-effective and has the potential to reduce HBV-associated morbidity and mortality.

## Introduction

Hepatitis B virus (HBV) infection is an important global health problem. Approximately 350 million people are chronically infected with the virus worldwide, 25% of whom will die from long-term sequelae, such as cirrhosis, liver failure and hepatocellular carcinoma (HCC), resulting in 600,000 to one million deaths annually [1,2]. Chronic HBV is generally asymptomatic for the first 30 to 40 years after infection. Individuals then typically present with symptoms related to long-term sequelae and generally respond poorly to treatment [3]. Morbidity and mortality from HBV can be reduced through screening individuals at risk for chronic HBV infection, and offering appropriately timed antiviral therapy in those found to be infected [4]. Furthermore, an effective HBV vaccine to protect those who are susceptible to HBV and prevent transmission has been available since the 1980s [5].

Over the past four decades, the majority of new migrants to Canada, as well as to most other major immigrant-receiving countries, have originated from countries with an intermediate or high prevalence of chronic HBV infection (hepatitis B surface antigen [HBsAg] seroprevalence ≥ 2%) [6]. Immigrants have increased mortality from chronic viral hepatitis and from HCC as compared to the Canadian-born and other native-born populations in immigrant-receiving countries, likely due to undetected chronic hepatitis B infection acquired in their countries of origin [7-14]. As a result, organizations such as the Centers for Disease Control and Prevention (CDC) and the Canadian Collaboration for Immigrant and Refugee Health (CCIRH) have recently recommended that 1) immigrants from areas where HBV prevalence is ≥ 2% be screened for chronic HBV, and 2) those found susceptible be vaccinated [4,15]. Despite this, there are currently no routine immigrant screening programs for chronic HBV infection or targeted catch-up vaccination programs before or after arrival in Canada and most other host countries [4,15-17]. Previous cost-effectiveness studies have shown that screening migrants for HBV was at least moderately cost-effective [18-20]. However, there were large variations in model assumptions, such as the timing of screening, age and origins of the target populations, estimated treatment compliance and economic perspective used.

To identify the optimal strategy to decrease the burden of HBV in the immigrant population we conducted a cost-effectiveness analysis, from a societal perspective, of four HBV vaccination and screening strategies for a representative cohort of new adult immigrants upon arrival to Canada, taking into consideration immigrant’s age and region of origin.

## Materials and Methods

### Intervention Strategies

A decision-analysis tree, which incorporated Markov processes to represent the natural history of HBV disease, was developed using TreeAge Pro 2011 (TreeAge Inc., Williamstown, MA) to evaluate the cost-effectiveness of four screening and vaccination strategies in an incoming cohort of new immigrants who were all assumed to be unaware of their HBV infection status and asymptomatic if chronically infected. These strategies were compared to the status quo of no targeted screening or vaccination for new Canadian immigrants.

#### The four strategies evaluated in this analysis were

**Figure 1:**
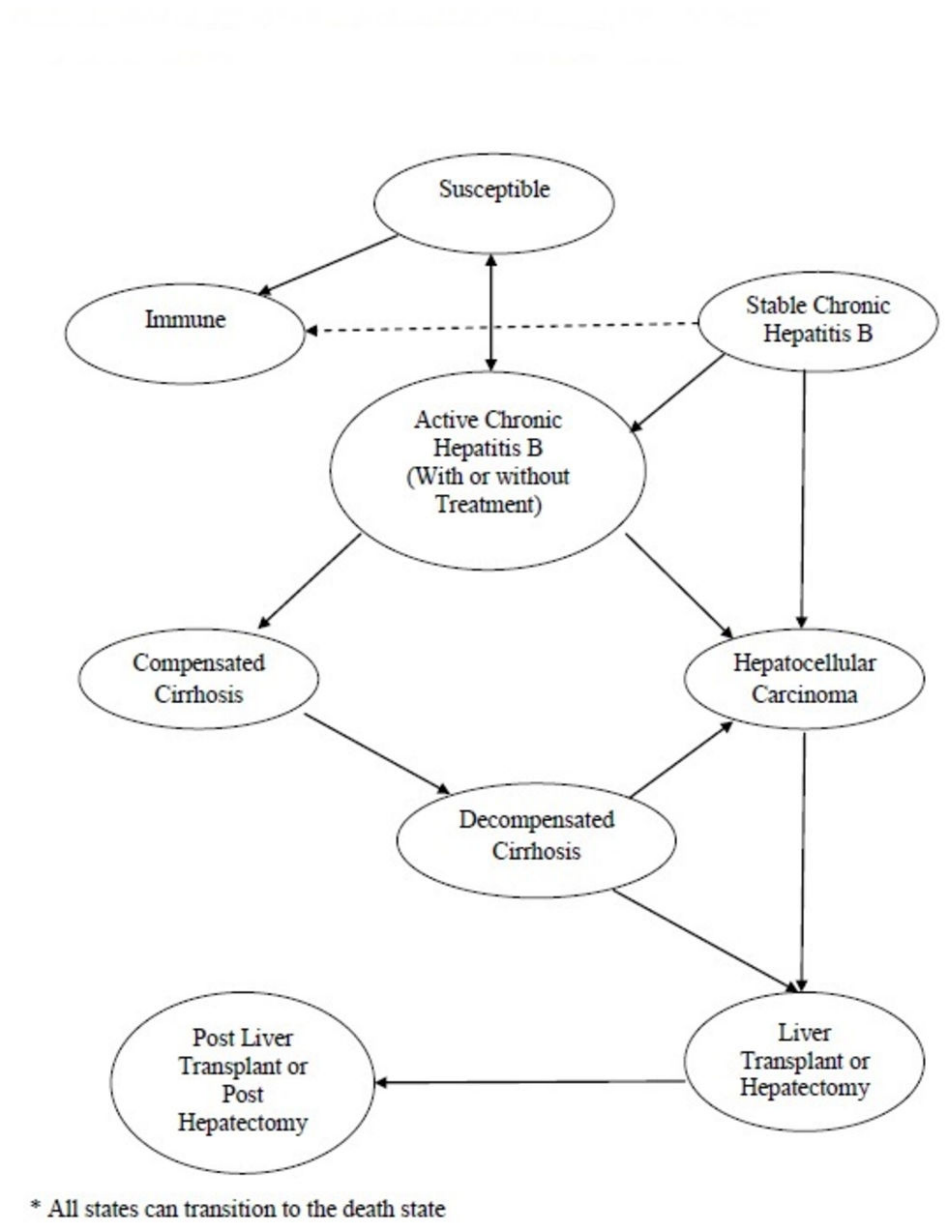
Markov model for the natural history of hepatitis B infection. Note: Every year individuals can transition to different health states (straight arrows) or can remain in their current health state. All health states can transition to an absorbing death state (not shown). Transitions occur annually until death. Immigrants enter the model based on their compliance with one of the interventions being offered.

**Figure 2:**
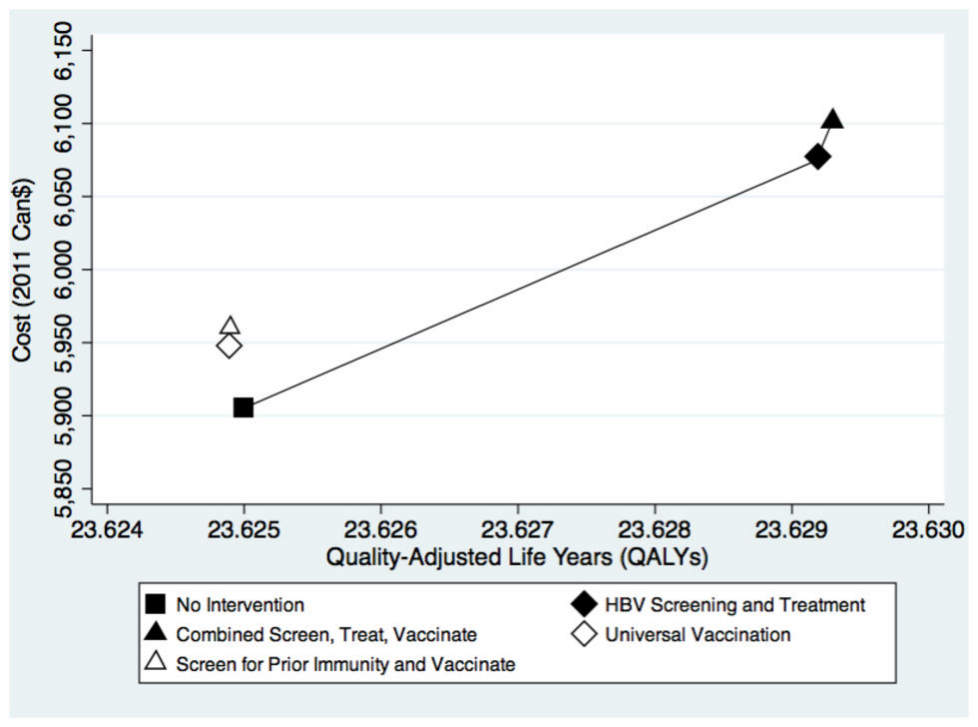
Cost-effectiveness plane comparing each interventions and the status quo for the base-case analysis. The graph plots the average cost in Canadian dollars of the various strategies against the average quality-adjusted life years experienced by the hypothetical cohort. The slope between the points of the undominated strategies (filled shapes) corresponds to the incremental cost-effectiveness ratio.

**Figure 3:**
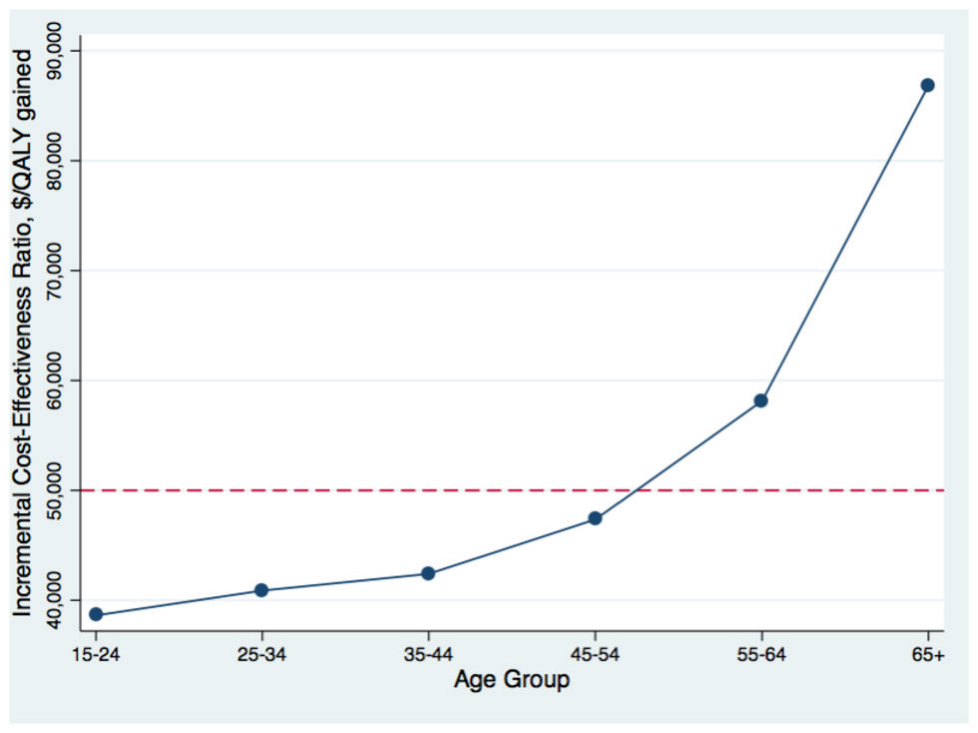
Change in the cost-effectiveness of the HBV screen and treat strategy by immigrant age group.

**Figure 4:**
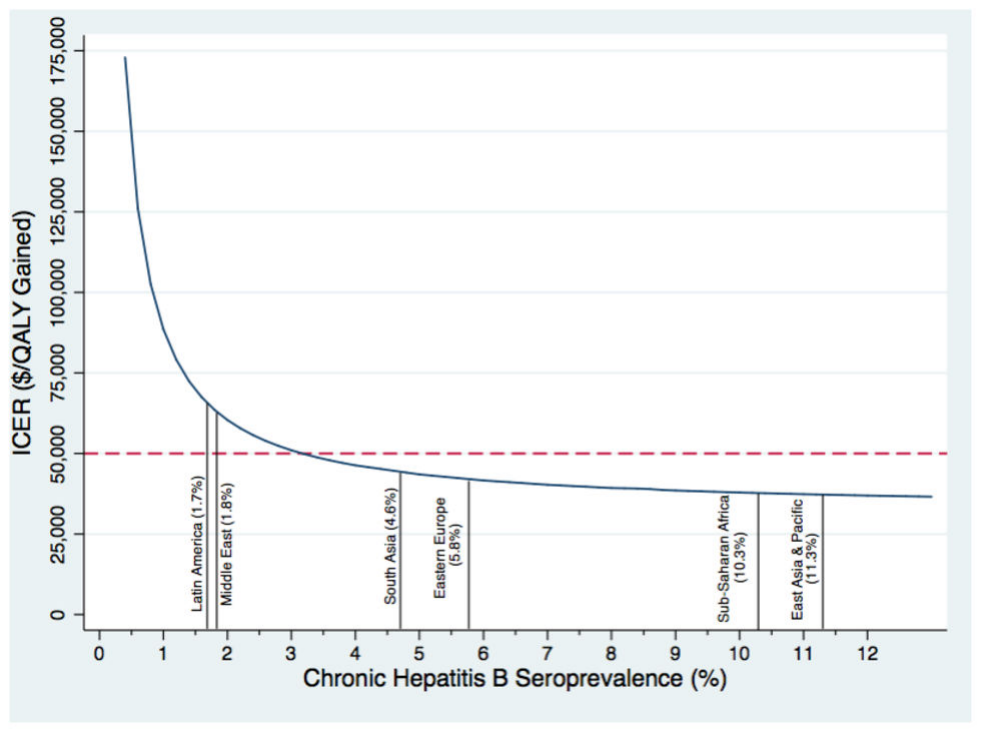
Variation in the cost-effectiveness of the HBV screen and treat strategy by immigrant seroprevalence. Note: The seroprevalence from a recent systematic review and meta-analysis is shown for the six major immigrant-source regions to Canada.

a. *Universal* *vaccination*: Immigrants are offered the three-dose vaccine series for HBV, without any serologic testing to determine who is already infected or immune, during their first year, after arrival in Canada. Those who are already infected do not receive any benefit from immunization, regardless if they complied with the intervention or not, and would proceed normally through the natural history of HBV disease. Those who are susceptible will generate protective antibodies according to their compliance with receiving one, two or three doses of the vaccine (see Figure 1 in Text S1).
b. *Screening* *for* *prior* *immunity and vaccination*: Immigrants are offered serologic testing for only hepatitis B surface antibody (anti-HBs) to determine who has prior immunity to HBV. Those found to have no serologic evidence of immunity are offered the three-dose vaccine series for HBV. Those who decline serologic testing and those found to be immune are not offered immunization. Similarly, those who are already chronically infected and accept the vaccine will receive no benefit from immunization (see Figure 2 in Text S1).
c. *Chronic* *HBV* *screening and treatment*: Immigrants are offered serologic testing for chronic HBV only. Those found to be positive for HBsAg will be referred to visit a liver specialist for further investigations and to determine the need for antiviral therapy. If treatment is indicated, then immigrants will be offered treatment with a first-line nucleoside analogue [3].Those immigrants who are found not to require antiviral therapy will be followed up annually to determine if subsequent treatment is needed (see Figure 3 in Text S1).
d. *Combined* *screening* *for* *chronic* *HBV and prior* *immunity*: Immigrants are offered serologic testing for both anti-HBs and HBsAg. As above, those found to be chronically infected are referred to a liver specialist. Those found to be susceptible are offered the three-dose vaccination series. Those who decline initial serologic testing are not offered immunization and are not referred to a liver specialist (see Figure 4 in Text S1).

### Model Structure

The population of interest was a hypothetical cohort of 250,000 new Canadian immigrants, representing the population of landed immigrants who have settled in Canada each year during the last decade [21]. For the time horizon, immigrants were followed until death. At entry, all immigrants were assumed to be in one of four mutually-exclusive underlying HBV health states: susceptible, immune, stable chronic infection or active chronic infection. We assumed that immigrants were otherwise healthy and did not arrive with cirrhosis or HCC [18]. After accepting or declining the intervention being offered during their first year, immigrants entered the Markov model based on the outcome of the intervention. For example, those who initially arrived susceptible and accepted to be vaccinated would enter the Markov model in the immunized state, if they developed an immune response to the vaccine. Within the Markov model, subjects can proceed to develop compensated cirrhosis, decompensated cirrhosis, HCC, undergo a liver transplant or hepatectomy, or die from these illnesses or die from other non HBV-related causes (Figure 1). The annual probabilities of transitioning between states in the model were obtained from cohort studies and from other cost-effectiveness analyses and systematic reviews (see Appendix Table 1 in Text S2). The model was validated by comparing estimated age-specific HCC mortality rates with data from the GLOBOCAN cancer registry (See Appendix Figure 1 in Text S2) [22]. 

**Table 1:** Costs and quality-adjusted life years of hepatitis B strategies in a cohort of Canadian immigrants.

**Strategy**	**Base-Case Mean cost per immigrant, two thousand and eleven Can$ ^a^**	**Base-Case Mean QALYs per immigrant ^a^**	**Base-Case Incremental cost-effectiveness ratio, $ per QALY gained ^b^**	**Age-Adjusted Mean cost per immigrant, two thousand and eleven Can$ ^a^**	**Age-Adjusted Mean QALYs per immigrant ^a^**	**Age-Adjusted Incremental cost-effectiveness ratio, $ per QALY gained ^b^**
No intervention	5,905	23.6250	Reference	5,429	21.7463	Reference
Universal vaccination	5,947	23.6249	Dominated	5,472	21.7462	Dominated
Screen for prior immunity and vaccinate	5,960	23.6249	Dominated	5,485	21.7462	Dominated
Chronic HBV screening and treatment	6,077	23.6292	40,880	5,599	21.7502	43,590
Combined screening, treatment and vaccination	6,101	23.6293	437,335	5,623	21.7503	243,400

Base-case was a 30-year old immigrant who was offered one of the screening and/or vaccination strategies or no intervention. Age-adjusted estimates were standardized to the age-distribution of new Canadian immigrants. Costs and QALYs discounted at a rate of 3% per year.

^a^ The average cost and quality-adjusted life years realized by each individual in the cohort.

^b^ Incremental cost-effectiveness ratio = difference in cost / difference in quality-adjusted life years gained.

HBV = hepatitis B virus.

QALYs = quality-adjusted life years.

Immigrants who had a *stable chronic* infection were assumed not to be candidates for antiviral treatment, as we considered them to have normal liver enzymes levels with low levels of circulating HBV DNA, and to be hepatitis B e-antigen (HBeAg) negative [3]. Immigrants who had an *active chronic* infection were assumed to be candidates for antiviral therapy as we considered them to have elevated liver enzymes, high levels of circulating HBV DNA, and were HBeAg positive, indicating active viral replication [23]. This dichotomous classification for chronic HBV infection was used to simplify the different combinations of clinical characteristics, such as viral load and HBeAg status, which guide clinical management of patients with chronic HBV [3]. Based on the relatively young age that immigrants develop chronic infections in HBV endemic countries and the fact that most of these perinatally acquired chronic infections can remain in an immune-tolerant phase for over twenty years [24], we assumed that 50% of chronically infected immigrants would have stable chronic infection and 50% would have active chronic infection upon arrival [20]. This assumption was varied in a sensitivity analysis to determine how it would affect our results. 

Susceptible individuals can become chronically infected, after landing in Canada, if they acquire an acute infection and fail to clear the virus. We assumed that the annual risk of infection in susceptible adults was 4.8 per 100,000 people among 30 to 39 year-olds, and 3.1 per 100,000 people among those ≥ 40 years [25]. We used rates from the general Canadian population, as incidence rates of acute HBV infection were unavailable for Canadian immigrants. The rates in the Canadian population were similar to reported rates in the U.S. foreign-born population, after accounting for under-reporting due to the often-asymptomatic nature of acute HBV infections in adults [26]. 

We estimated that 5% of adult immigrants who acquire an acute HBV infection will subsequently develop chronic hepatitis B [2,27]. All new cases of chronic HBV acquired in adult immigrants were assumed to be active chronic infections because most acquired cases in adults are associated with elevated levels of liver enzymes and circulating viral DNA [24]. 

### Seroprevalence Estimates

 To determine the initial proportions of immigrants who arrived chronically infected or with prior immunity, we used data from a systematic review and meta-analysis that reported region of origin-specific HBsAg and anti-HBs seroprevalence estimates in immigrants and refugees [28]. These seroprevalence estimates were weighted by the proportion of Canadian immigrants, arriving between 2001 and 2006, from each of the same world regions to determine an overall average HBsAg and anti-HBs seroprevalence estimate for newly-arriving immigrants in the model [29]. Accordingly, for our base-case models, we estimated that 6.5% of the hypothetical immigrant cohort would be chronically infected upon arrival and 32% would have prior immunity to HBV (see Appendix Table 1 in Text S2). 

### Model Assumptions

 For each of the strategies involving serologic testing, we assumed that 70% of the population would accept to be screened. Similarly, we assumed that 70% would accept the first dose of the HBV vaccine, if offered, and of these 85% and then 90% would receive second and third doses, respectively, given that they received the previous dose. As no empirical data on compliance with voluntary serologic testing and vaccination uptake in immigrant populations exists, these estimates are our own assumptions and we examined the sensitivity of these, and all other assumptions in our model (see Sensitivity Analysis below). Vaccine immunogenicity following each of the three doses was 42.5%, 75% and 90%, respectively, and was assumed to provide lifelong immunity [30]. We assumed that acceptance of serologic testing or immunization was independent of an individual’s initial infection status. Since chronic HBV is often asymptomatic in adults and we assumed immigrants were unaware of whether they were susceptible, immune or infected, this assumption of independence is valid and is not expected to influence the validity of our model. To account for screening that occurs during routine clinical care, rather than during the targeted public health programs, we assumed that members of the cohort had a 2% annual probability of being serologically tested for chronic HBV infection after arriving in Canada. 

 For those found to be chronically infected after serologic testing, we assumed that 60% would agree to visit a liver specialist to assess the need for treatment [19]. If treatment is indicated because of an active chronic infection, we assumed that 75% would immediately initiate antiviral therapy. We assumed that antiviral therapy would reduce the risk of progressing from active chronic infection to compensated cirrhosis, HCC or death, by 50% [31]. 

### Direct and Indirect Cost Estimates

 All direct and indirect costs were calculated in Canadian dollars for the year 2011 and are summarized in Appendix Table 2 (see Text S2). Published costs from previous years were converted into two thousand and eleven dollars using the Consumer Price Index for health care goods and services [32]. Our economic evaluation was performed from a societal perspective and included direct medical costs attributed to acute and chronic HBV infections and their sequelae, as well as indirect costs, which included out-of-pocket costs to patients and their families, such as time taken by family members during palliative care for hepatocellular carcinoma. Productivity costs, measured by lost income due to death or disability from chronic HBV sequelae, were not included in cost-effectiveness calculations to avoid double counting of the impact of chronic sequelae already captured by health-state utilities [33]. 

Program costs related to serologic testing and visits to liver specialists were obtained from medical payment schedules from the Quebec and British Columbia ministries of health [34,35]. Vaccine costs were obtained through personal correspondence from the Montreal Public Health Department. Direct and indirect medical costs associated with an acute HBV episode, including time lost from work for hospital visits and care were incorporated into the model [36]. We estimated that the average daily gross wage for an adult immigrant was $142.50 ($19/hour for 7.5 hours), and that symptomatic acute infections would result in time off work [37].

 Direct health-care costs for chronic HBV and liver disease sequelae were obtained from a Canadian study that calculated the expected cost of care for patients with chronic HBV and resulting complications [38]. Antiviral treatment was assumed to cost $8,089 per year, which is the average between the annual cost of Tenofovir and Entecavir, two first-line antiviral medications for chronic HBV [39]. 

### Health-State Utilities

Chronic HBV infections and the long-term liver diseases that result from infection have a substantial impact on an individual’s quality of life. We used utility estimates for chronic HBV-related health states elicited from uninfected Canadian respondents to calculate quality-adjusted life years (QALYs) in the cost-effectiveness analysis (Appendix Table 3 in Text S2) [40]. All future costs and outcomes were discounted at an annual rate of 3% [33]. 

### Cost-Effectiveness Analyses

 We first calculated expected costs and quality-adjusted survival for each strategy. Strategies were compared by calculating their incremental cost-effectiveness ratio (ICER), which is defined as the additional health benefit of an intervention, measured in QALYs gained, with the next least costly undominated strategy [41]. Strategies costing < $50,000 per additional QALY gained were considered to be cost-effective. Base-case analyses assumed all cohort members were 30 years old and the underlying HBV seroprevalence was 6.5%. 

### Sensitivity Analysis

We examined how ICERs for the strategies varied by considering cohorts with different seroprevalence levels and age structures, including one with the same age distribution as current immigrants to Canada. One-way and two-way sensitivity analyses were performed to determine how key assumptions regarding compliance estimates and cost parameters influenced base-case results. One-way sensitivity analyses were represented with a tornado diagram which shows how our base-case estimates for ICERs would change as a single parameter is varied. We conducted a two-way sensitivity analysis for treatment cost and treatment efficacy to examine how our estimates would change if newer, more effective, or inexpensive antivirals were utilized. A probabilistic sensitivity analysis (PSA), using 10,000 Monte Carlo simulations, was conducted to simultaneously assess uncertainty around all key parameters [42,43]. Transition probabilities and compliance estimates for the PSA were assumed to follow a beta distribution and costs followed a gamma distribution [44]. Results from the PSA were assessed with a cost-effectiveness acceptability curve, which shows the probability that a strategy is cost-effective given several ICER thresholds.

## Results

### Base-Case Analysis

In the base-case analysis, screening for chronic HBV and offering appropriately timed antiviral treatment was the most cost-effective strategy (Table 1 and Figure 2). The incremental cost per additional QALY gained for this strategy compared to no intervention was $40,880. The combined chronic HBV screening and vaccination strategy was estimated to cost $437,335 for each additional QALY gained compared to chronic HBV screening alone. Both strategies involving vaccination alone, with or without prior serologic testing for immunity, were dominated by the HBV screening and treatment strategies, as the former strategies cost more and yielded fewer QALYs than the no intervention strategy. When we considered a cohort which had a similar age-distribution as a cohort of new Canadian immigrants, the ICER comparing the screen and treat strategy to no intervention increased slightly to $43,590 per QALY gained (Table 1). As in the base-case analysis, both vaccination only strategies were dominated, while the screen, treat and vaccination strategy was prohibitively expensive. 

The cost-effectiveness of the screen and treat strategy decreased as cohort age was increased in the model (Figure 3). The ICER comparing the HBV screen and treatment strategy to no targeted intervention exceeded $50,000 per additional QALY gained for new Canadian immigrants aged 55 years old and older. Screening newly arriving immigrants from regions where the seroprevalence of chronic HBV infection was greater than 3% was found to be cost-effective, using this same threshold (Figure 4). For immigrants from low HBV endemic areas (HBsAg seroprevalence < 2%), such as Latin America and the Middle East, the screen and treat strategy was estimated to cost approximately $64,000 per additional QALY gained relative to the no intervention strategy. For immigrants from intermediate HBV endemic areas (HBsAg 2% to 8%), such as South Asia and Eastern Europe, the screen and treat strategy was estimate to cost less than $43,000 per additional QALY gained, and for highly HBV endemic areas (HBsAg > 8%), such sub-Saharan Africa and East Asia, the same strategy was estimated to cost less than $37,000 per additional QALY gained. The screen and treat strategy dominated the vaccination only, as well as the screen and vaccinate strategy, at every modeled seroprevalence level and age group. 

### Other Sensitivity Analyses

**Figure 5:**
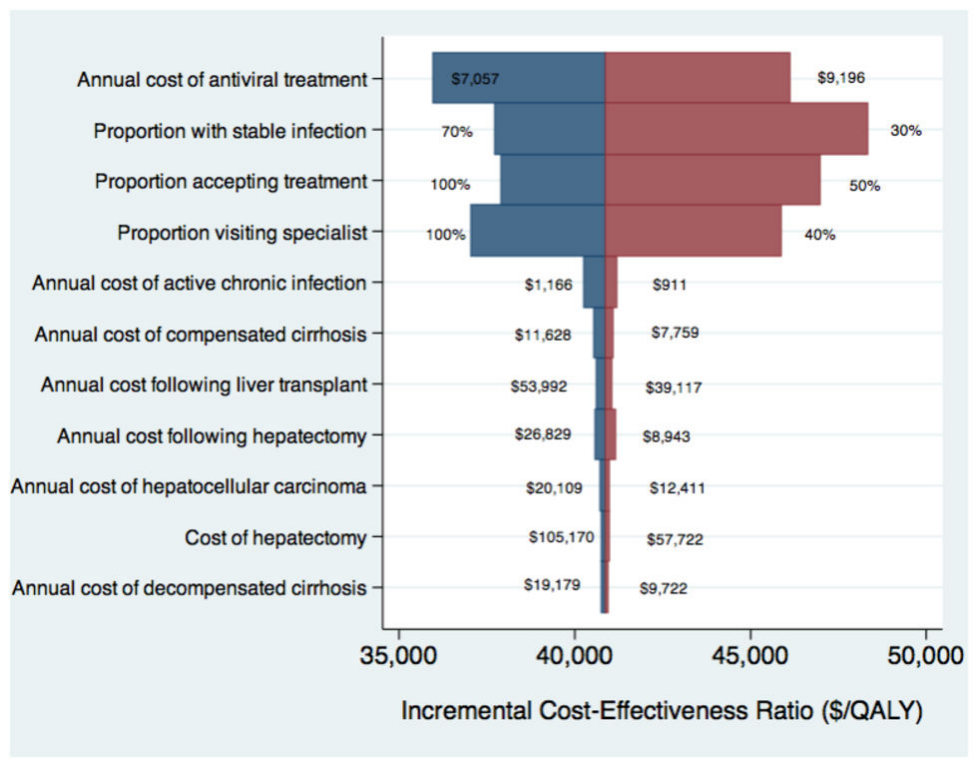
Tornado diagram for one-way deterministic sensitivity analysis.

The chronic HBV screening and treatment strategy remained the most cost-effective strategy in all one-way sensitivity analyses. The results of multiple one-way sensitivity analyses are summarized in Figure 5. Notably, the ICER for the HBV screening and treatment strategy increased from $37,728 to $48,343 when the estimate of the proportion of immigrants with stable chronic infection decreased from 70% to 30%. Results were sensitive to the proportion of immigrants complying with the various components of the interventions, although none of the model assumptions had a major impact on the main findings reported above. Specifically the cost-effectiveness of the HBV screening and treatment strategy increased as the proportion of those with chronic infection visited a specialist, accepted or complied with treatment. 

**Figure 6:**
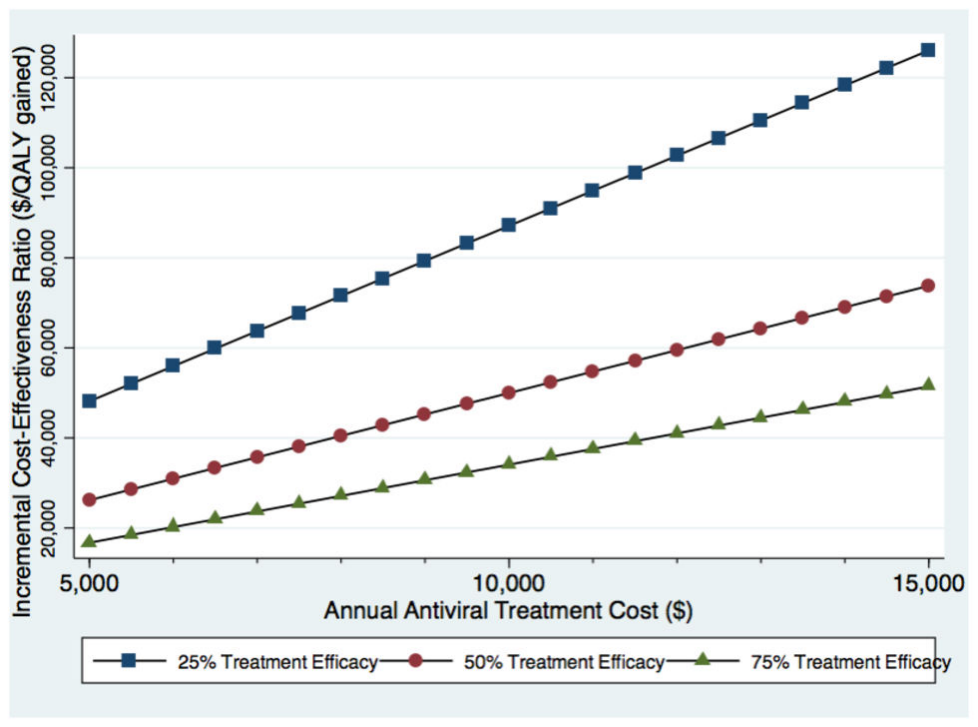
Variation in the cost-effectiveness of the HBV screen and treat strategy by antiviral treatment cost and efficacy.

 Results were sensitive to the cost of antiviral therapy and treatment efficacy. In a two-way sensitivity analysis of the screen and treat strategy, it was found that the incremental cost per additional QALY gained exceeded $50,000 when the cost of treatment was ≥ $10,000 per year, using the base-case treatment efficacy of 50% (Figure 6). If treatment efficacy increased to 75%, this strategy remained relatively cost-effective, even at very high treatment costs. The probabilistic sensitivity analysis produced no change in the ranking of strategies and did not substantially change the ICER estimates. At a willingness-to-pay threshold of $50,000 per QALY gained, the chronic HBV screening strategy had a 78% chance of being the most cost-effective strategy (See Appendix Figure 2 in Text S2). At a threshold of $80,000 per QALY gained, it was found that the chronic HBV screening strategy had a 99% chance of being the most cost-effective. 

## Discussion

The results of our study suggest that targeted screening of immigrants for chronic HBV infection, followed by appropriately timed antiviral therapy is the most cost-effective option to decrease HBV-associated morbidity and mortality in immigrants. From a societal perspective, it was estimated that this intervention would cost $40,880 per additional QALY gained as compared to no intervention in Canada. The cost-effectiveness of screening for chronic infection remained < $50,000 per additional QALY gained when HBV seroprevalence was as low as 3%. The CDC and the CCIRH have recently recommended HBV screening for all immigrants from countries where the HBV prevalence is ≥ 2% which would include all world regions with the exception of most countries in Latin America, the Middle East and Western Europe [4,15]. Our results support this recommendation given that immigrants from all other world regions have a mean seroprevalence of ≥ 2% [28]. Although our findings differ slightly from the recommendations made by the CDC and the CCIRH, the benefits of screening an entire cohort of immigrants for chronic HBV infection is, nonetheless, substantial. Furthermore, our model estimates that the targeted screening program for chronic HBV infection in all immigrants upon arrival would results in an additional 1,675 productive life-years gained for every 250,000 immigrants screened, relative to no target screening. As a result, the findings of this cost-effectiveness analysis strongly support the public health recommendations made by the CDC and the CCIRH [4,15]. 

The benefit of screening for chronic HBV in immigrants is that it identifies individuals with asymptomatic chronic HBV infection acquired in their countries of origin, who, if left undetected are at increased risk of progression to HBV-related sequelae, such as liver cirrhosis and HCC. With the timely identification and treatment of infected individuals the risk of developing these long-term sequelae can be reduced [23]. Each case of compensated cirrhosis and HCC costs the Canadian health-care system more than $9,000 and $15,000 per person every year, respectively. Although interventions that involved immunization have the potential to eliminate future HBV infections, they were dominated by those strategies that involved screening for chronic infection. This is due to the large costs associated with vaccinating an entire cohort, relative to the small absolute decrease in morbidity and mortality that would occur from preventing sequelae from a small number of new chronic infections that actually develop in the adult population. Immunizing adult immigrants has less of an impact than vaccinating children, since only approximately 5% of adults develop chronic infection after an acute episode, whereas this risk is as high as 90% in the neonatal period and 25-50% in young children [45]. Furthermore, vaccination interventions have no effect on reducing morbidity and mortality among individuals already chronically infected with HBV. 

 There have been three previous cost-effectiveness analyses that have addressed HBV screening or vaccination in immigrant populations [18-20]. Hutton et al. found that screening people of Asian and Pacific Islander descent would be cost-effective ($36,000 per additional QALY gained [2006 $USD]), compared to no screening and would reduce HBV-related mortality by 10% [18]. This study, however, only considered Asian Americans, which are only one high-risk immigrant group for liver disease and did not include indirect medical costs in their analysis. Veldhuijzen et al. found that a one-time screening program for immigrants to the Netherlands would be cost-effective (€9,000 per additional QALY gained [2008 €EUR]). This study considered varying levels of compliance with the intervention, but examined only direct medical costs and did not examine the sensitivity of their estimates with respect to age and seroprevalence level [19]. Wong et al. examined the cost-effectiveness of screening all Canadian immigrants for HBV and found it to be moderately cost-effective ($45,000 per additional QALY gained), although they assumed 100% compliance with the screening intervention [20]. The results of the present cost-effectiveness analysis, which estimated that screening new Canadian immigrants would cost $40,880 per additional QALY gained, are similar to the results obtained by Hutton et al. and agree in general with the results in the other two analyses. Our analysis adds to the previous published cost-effectiveness study by estimating the cost-effectiveness of HBV prevention strategies from a societal perspective, which included indirect costs, as well as a calculation of the seroprevalence threshold of chronic HBV infection above which it is worthwhile to perform HBV screening in immigrants.

In our study, we used a simplified model of the natural history of HBV, which did not account for HBeAg status, HBV DNA viral load, or alanine aminotransferase levels as in two earlier studies [20,46]. Instead, we partitioned chronic infection into stable and active chronic HBV states. Both our simpler approach, and the more elaborate classification scheme used previously, estimate that approximately 50% of immigrants diagnosed with chronic HBV infection are eligible for treatment. We made several assumptions regarding compliance with screening, vaccine uptake, specialist visits, and treatment recommendations, in the absence of clinical or epidemiologic studies documenting these parameters in immigrant populations. However, our estimates for these parameters were similar to those used in the Netherlands [19]. Our results also demonstrated that the analysis was sensitive to compliance with serologic testing, vaccination, antiviral therapy and follow-up visits to a liver specialist highlighting certain factors that need to be carefully considered when developing and assessing HBV screening and vaccination programs in this population [47].

 The challenges of implementing a widespread HBV screening program have been highlighted in the recent U.S. Institute of Medicine Report addressing prevention and control of hepatitis B and C [48]. Barriers to screening are a particularly important issue and include lack of knowledge and awareness about HBV infection and screening among patients, health care and social service providers and policy makers. It has been recommended that culturally sensitive outreach programs that promote awareness about hepatitis B infection in the community, as well as the potential benefits of screening and vaccination strategies, be integrated with health-care services that serve immigrant populations [49]. 

In Canada, it is estimated that less than 50% of immigrants have undergone HBV screening [16]. Similarly, the United States has reported low rates of HBV screening and vaccine uptake in immigrant populations, particularly among Asian and Pacific Islanders [50-52]. There is presently no program of routine health-care for adult immigrants after arrival in Canada and therefore broad-based community screening of all immigrants would require new infrastructure as well as educational efforts targeting both immigrants and health care providers for the need for HBV screening. A review of screening programs in the United States identified several community screening programs serving foreign-born populations, which were also providers of immunization, education and specialist referrals for the population served [53], however they were primarily community-based and were unable to screen a large number of high-risk immigrants. Screening at the time of arrival, when immigrants first come into contact with the health-care system may overcome some of these barriers, however programs to screen the large number of immigrants at risk for chronic HBV still need to be developed. 

## Conclusions

 Screening for chronic HBV in adult immigrants soon after arrival was found to be reasonably cost-effective, particularly in younger immigrants and those from most intermediate and also high HBV-endemic countries. To ensure immigrants will benefit from these study results, policy makers need to be made aware of the importance of developing targeted programs to screen for chronic HBV and clinicians and immigrants need to be educated about the importance of screening for this virus. Introduction of targeted HBV screening for all immigrants has the potential to substantially reduce HBV-associated morbidity and mortality in this population. 

## Supporting Information

Text S1 **TreeAge Decision-Analysis Models.**
(PDF)

Text S2 **Supplemental Information: Estimates, Costs, and Utilities.**
(PDF)
