# Supplementary figures and images for: Hepatitis B Screening and Vaccination Strategies for Newly Arrived Adult Canadian Immigrants and Refugees: A Cost-Effectiveness Analysis
Source: PLoS One. 2013 Oct 18;8(10):e78548. doi: 10.1371/journal.pone.0078548 (PMC3799697; doi:10.1371/journal.pone.0078548)

Figure 1

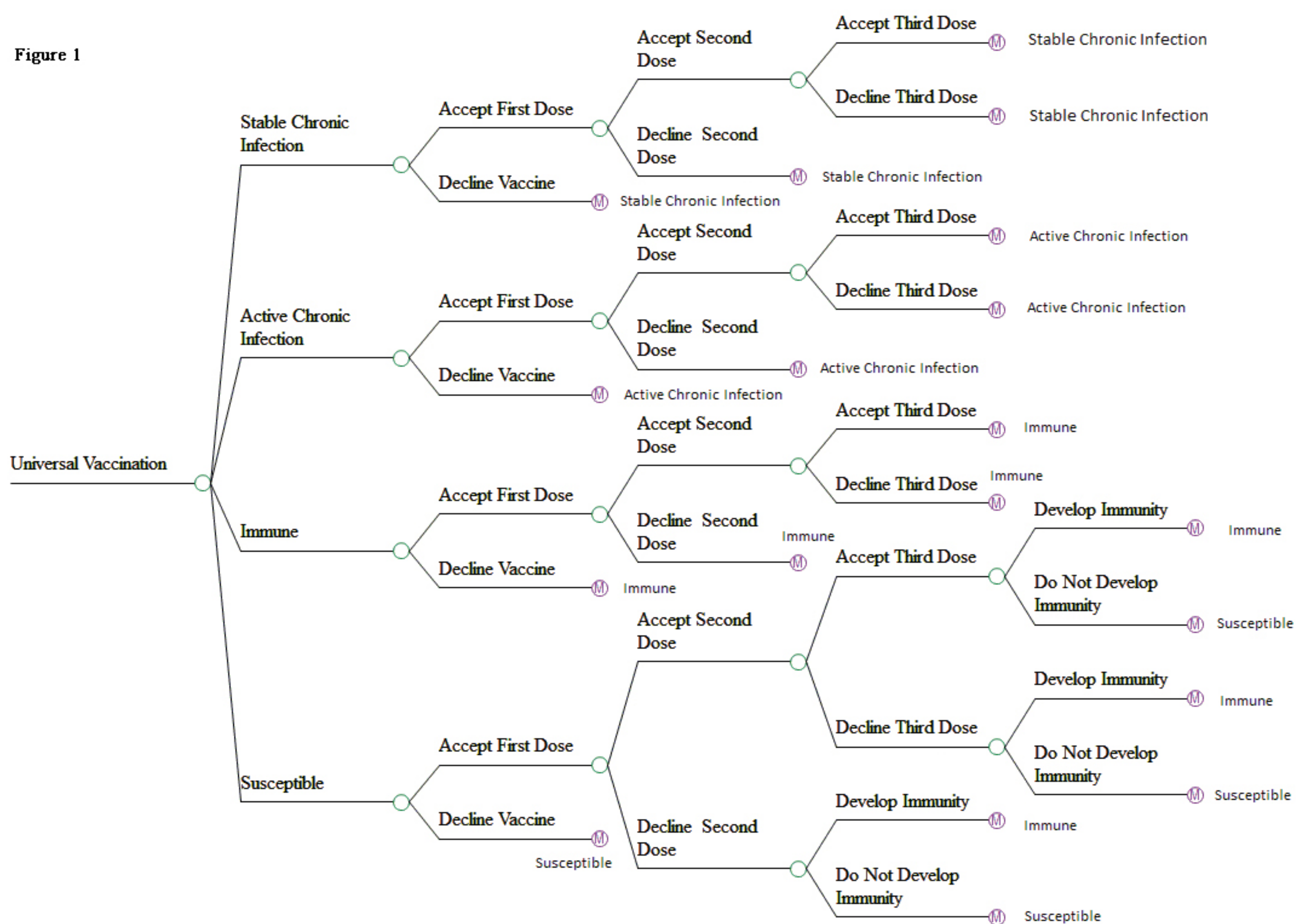

**Figure 2**

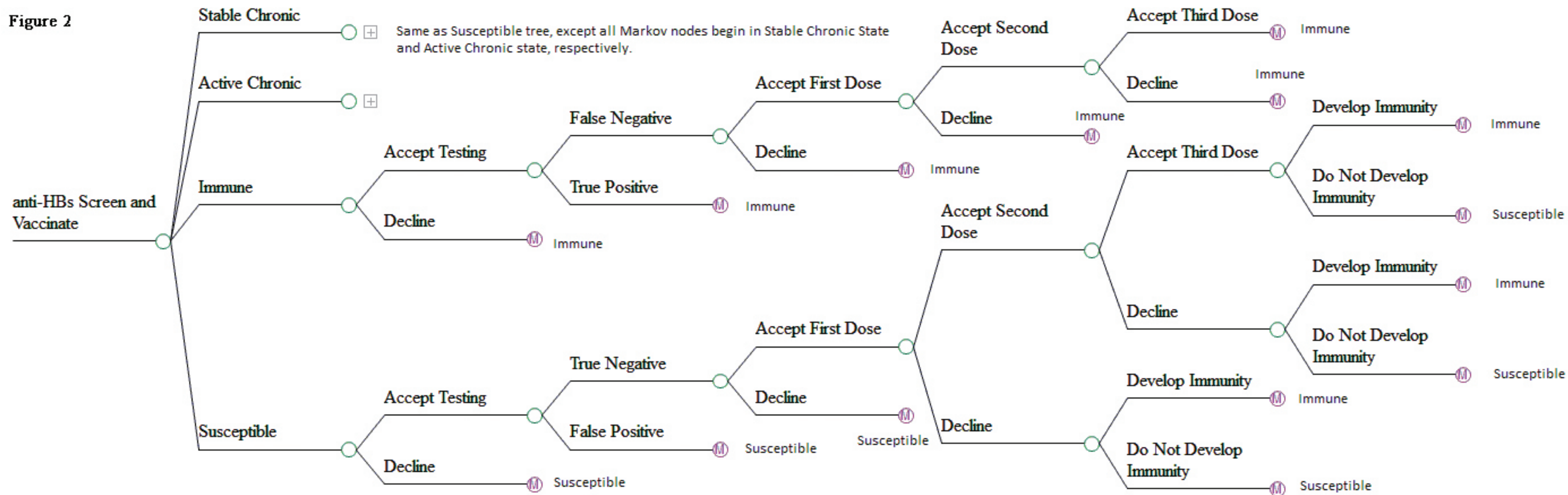

Figure 3

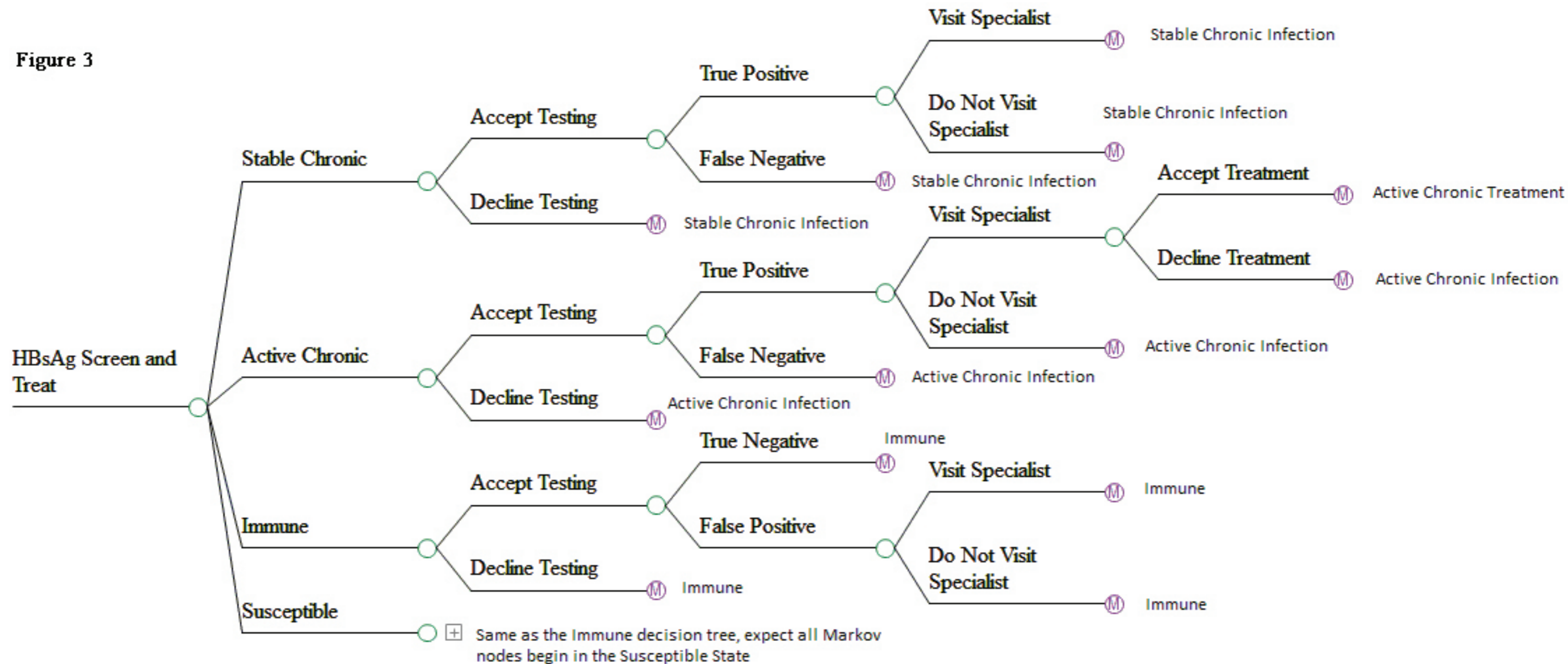

Figure 4

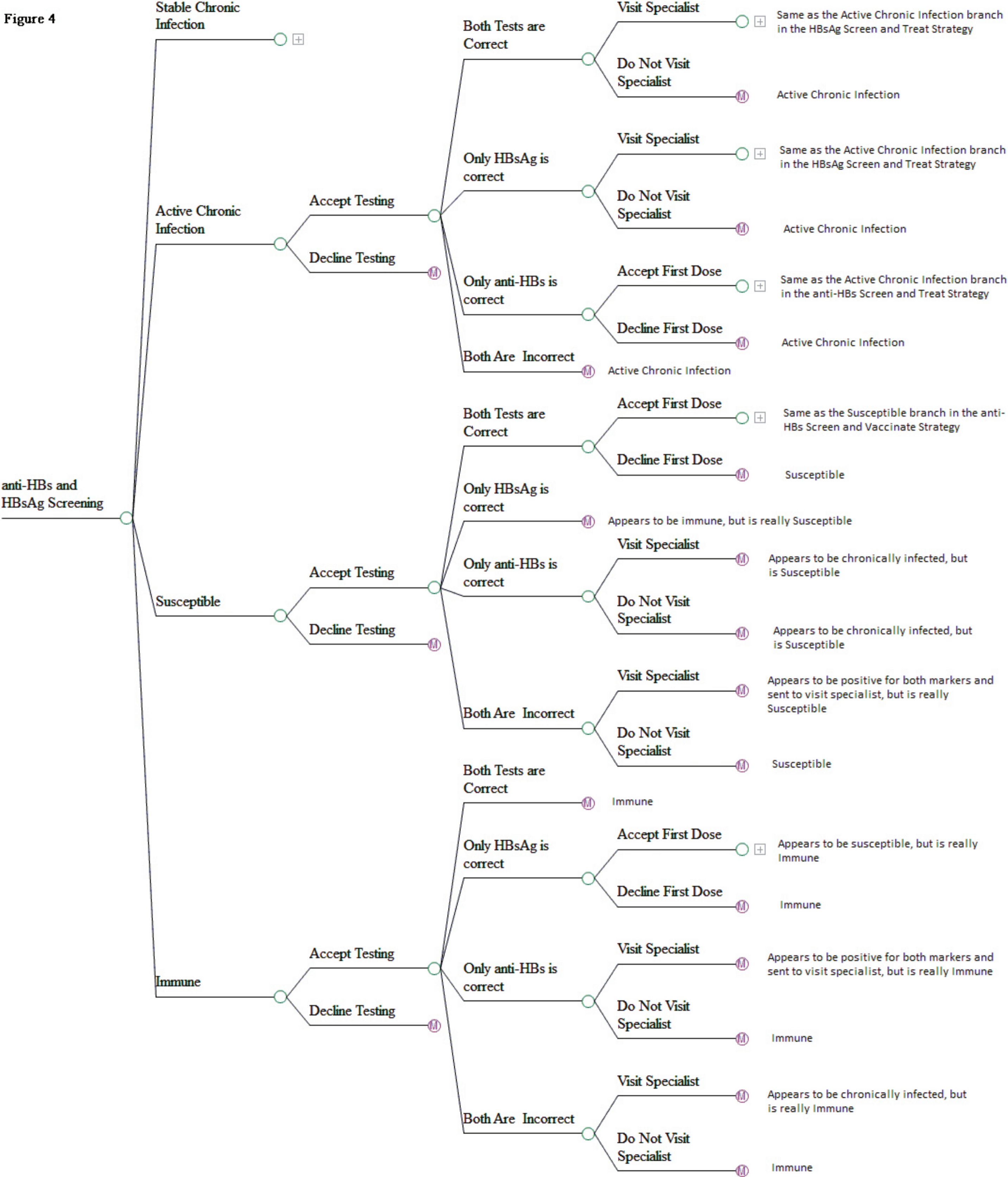

Supplement: Text S1 — TreeAge Decision-Analysis Models. (PDF) [file pone.0078548.s001.pdf]
