# Supplementary material for: Hepatitis B Screening and Vaccination Strategies for Newly Arrived Adult Canadian Immigrants and Refugees: A Cost-Effectiveness Analysis
Source: PLoS One. 2013 Oct 18;8(10):e78548. doi: 10.1371/journal.pone.0078548 (PMC3799697; doi:10.1371/journal.pone.0078548)
Supplement: Text S2 — Supplemental Information: Estimates, Costs, and Utilities. (PDF) [file pone.0078548.s002.pdf]

Text S2: Supplemental Material for Manuscript

Appendix Table 1. Seroprevalence estimates, intervention compliance and transition probabilities for hepatitis B screening and vaccination strategies.

Variable	Base-case estimate	Range for sensitivity analysis	Source
**Hepatitis B Prevalence**			
Chronic HBV infection	6.52%		Calculated from [1]
Prior HBV immunity	32.35%		Calculated from [1]
Probability that a chronic HBV infection is stable (*treatment not required*)	50%	30% – 70%	[2-4]
**Compliance with Interventions**			
Serologic testing	70%	40% – 100%	[5]
1st dose of vaccine	70%	40% – 100%	Assumed
2nd dose of vaccine, given 1st dose	85%	50% – 100%	Assumed
3rd dose of vaccine, given 2nd dose	90%	50% – 100%	Assumed
Visit liver specialist, if chronically infected	60%	40% – 100%	[6]
Accept antiviral treatment, if indicated	75%	50% – 100%	Assumed
**Intervention Characteristics**			
HBsAg testing sensitivity	99.5%		[7]
HBsAg testing specificity	99.5%		[7]
anti-HBs testing sensitivity	100%		[8]
anti-HBs testing specificity	97.9%		[8]
Vaccine immunogenicity, one dose	42.5%	30% – 55%	[9]
Vaccine immunogenicity, two doses	75%	62.5% – 87.5%	[9]
Vaccine immunogenicity, three doses	90%	80% – 100%	[9]
Antiviral treatment efficacy	50%	25% – 75%	[10]
**Annual Acute Hepatitis B Transitions**			
Risk of Infection			
30-39 years of age	0.0048%		[11]

≥ 40 years of age	0.0031%		[11]
Symptomatic infection	40%	30% – 50%	[11]
Hospitalization, given symptomatic infection	40%	12% – 50%	[5,12]
Fulminant hepatitis	1.5%	1% – 5%	[5,13]
Mortality from fulminant hepatitis	85%	53% – 100%	[13,14]
Chronic infection	5%	1% – 10%	[15,16]
**Annual Chronic Hepatitis B Transitions**			
Risk of death from other causes	Age Specific		[17]
Probability of HBsAg seroconversion	0.5%	0% – 1%	[18]
**Stable Chronic Infection**			
To active chronic infection	1.8%	0.84% – 2.7%	[19]
To HCC	0.34%	0.11% – 0.5%	[5,19,20]
To death	0.72%	0.38% – 0.93%	[19,21]
**Active Chronic Infection**			
To compensated cirrhosis	2.4%	0.7% – 3.8%	[5,22,23]
To HCC	1.1%	0.27% – 2.77%	[19,20,24]
To death	1.0%	0.25% – 1.5%	[19,21]
To antiviral treatment	50%	25% – 75%	Assumed
**Active Chronic Infection Treatment**			
To defaulting treatment	10%	0% – 20%	Assumed
To compensated cirrhosis	1.2%	0.6% – 2.8%	Assumed 50% treatment efficacy
To HCC	0.55%	0.28% – 0.83%	Assumed 50% treatment efficacy
To death	0.5%	0.25% – 0.75%	Assumed 50% treatment efficacy
**Compensated Cirrhosis**			
To decompensated cirrhosis	5.0%	3.24% – 7.0%	[5,25]

To HCC	5.0%	3.0% – 6.6%	[19,20,26]
To death	3.0%	1.3% – 4.8%	[5,25,27]
**Decompensated Cirrhosis**			
To HCC	6.3%	3.0% – 7.0%	[5,20,28]
To liver transplant	10%	0% – 40%	Assumed
To death	22.5%	9.9% – 31.4%	[5,19,25]
**Hepatocellular Carcinoma**			
To liver transplant	5.9%	5% – 40%	[29,30]
To hepatectomy	24.5%	15% – 45%	[29,30]
To death	35%	8.1% – 54.5%	[19,31,32]
**Liver Transplant 1st Year**			
To death	9.2%	5% – 20%	[33]
**Post Liver Transplant**			
To death	5%	1% – 15%	[33]
**Hepatectomy 1st Year**			
To death	6%	0% – 8%	[34]
**Post Hepatectomy**			
To death	12%	9% – 17%	[34]

Appendix Table 2: Program costs for hepatitis B prevention and treatment strategies.

Variable	Base-case estimate	Range for sensitivity analysis	Source
**Discount rate, costs and utilities**	3%	0% – 5%	[35]
**Program costs**			
Vaccine price per dose	$10.07		[36]
Vaccine administration per dose	$15		[5]
Cost of hepatitis B Surface antigen test (chronic HBV screen)	$6.00		[37]
Cost of hepatitis B surface antibody test	$8.00		[37]
Laboratory administration	$45		Assumed
Liver specialist visit	$176.30		[38]
Liver specialist testing and administration	$136.58		[39]
**Direct medical costs – Acute Infection**			
Symptomatic acute infection, no hospitalization	$375	$188 – $647	[40]
Symptomatic acute infection, hospitalization	$4,515	$2,372 – $11,148	[41]
Fulminant hepatitis	$18,044		[40]
**Direct annual medical costs – Chronic Disease**			
Active chronic infection related costs	$1,001	$911 – $1,166	[42]
Chronic HBV treatment: *Tenofovir or Entecavir*	$8,089	$7,057 – $9,196	[42,43]
Compensated cirrhosis costs	$9,279	$7,759 – $11,628	[42]
Decompensated cirrhosis costs	$13,137	$9,722 – $19,174	[42]
Hepatocellular carcinoma costs	$15,620	$12,411 – $20,109	[42]
1st year liver transplant costs	$115,907	$110,364 – $124,995	[42]
Post liver transplant costs	$44,734	$39,117 – $53,992	[42]
1st year hepatectomy costs	$80,397	$57,722 – $105,170	[44]
Post hepatectomy costs	$17,886	$8,943 – $26,829	[44]
**Indirect costs – Acute Infection**			
Symptomatic acute infection, no hospitalization, time lost from work (three days)	$427.50	$0 – $712.50	Assumed
Symptomatic acute infection,	$1,140	$660 – $1,800	Calculated

hospitalization, time lost from work (eight days)			from [41]
Fulminant hepatitis, time lost from work (thirty days)	$4,275		Assumed
**Indirect costs – Chronic Disease**			
Palliative Care for HCC-related death	$3,095	$0 – $5,000	[45]

Appendix Table 3. Health-state utilities for Markov model.

Utilities	Base-case estimate	Range for sensitivity analysis	Source
Susceptible	0.99	0.95 – 1.0	Assumed
Immune	0.99	0.95 – 1.0	Assumed
Stable Chronic Infection	0.95	0.9 – 1.0	Assumed
Active Chronic Infection	0.85		[46]
Hepatitis B Treatment	0.90	0.85 – 0.95	Assumed
Compensated Cirrhosis	0.83		[46]
Decompensated Cirrhosis	0.45		[46]
Hepatocellular Carcinoma	0.47		[46]
Liver Transplant (1st Year)	0.71		[46]
Post Liver Transplant	0.80		[46]
Hepatectomy (1st Year)	0.75	0.70 – 0.80	Assumed
Post Hepatectomy	0.85	0.80 – 0.90	Assumed

Appendix Figure 1: Model Validation Results

To validate the model, annual average age-specific mortality rates for HCC that were generated by observing the hypothetical cohort of immigrants as they progressed through the Markov model from age 40 to age 80 were compared to liver cancer mortality data obtained from the GLOBOCAN 2008 database [47]. For most age groups, estimates of HCC mortality from our model closely approximate GLOBOCAN data for mortality rates among the combined WHO regions of the Western Pacific, Southeast Asia and Sub-Saharan Africa, which are the three largest source regions of new immigrants to Canada. Our model, however, slightly underestimates mortality among 40-44 year olds, and overestimates mortality among those between 75-80 years old.

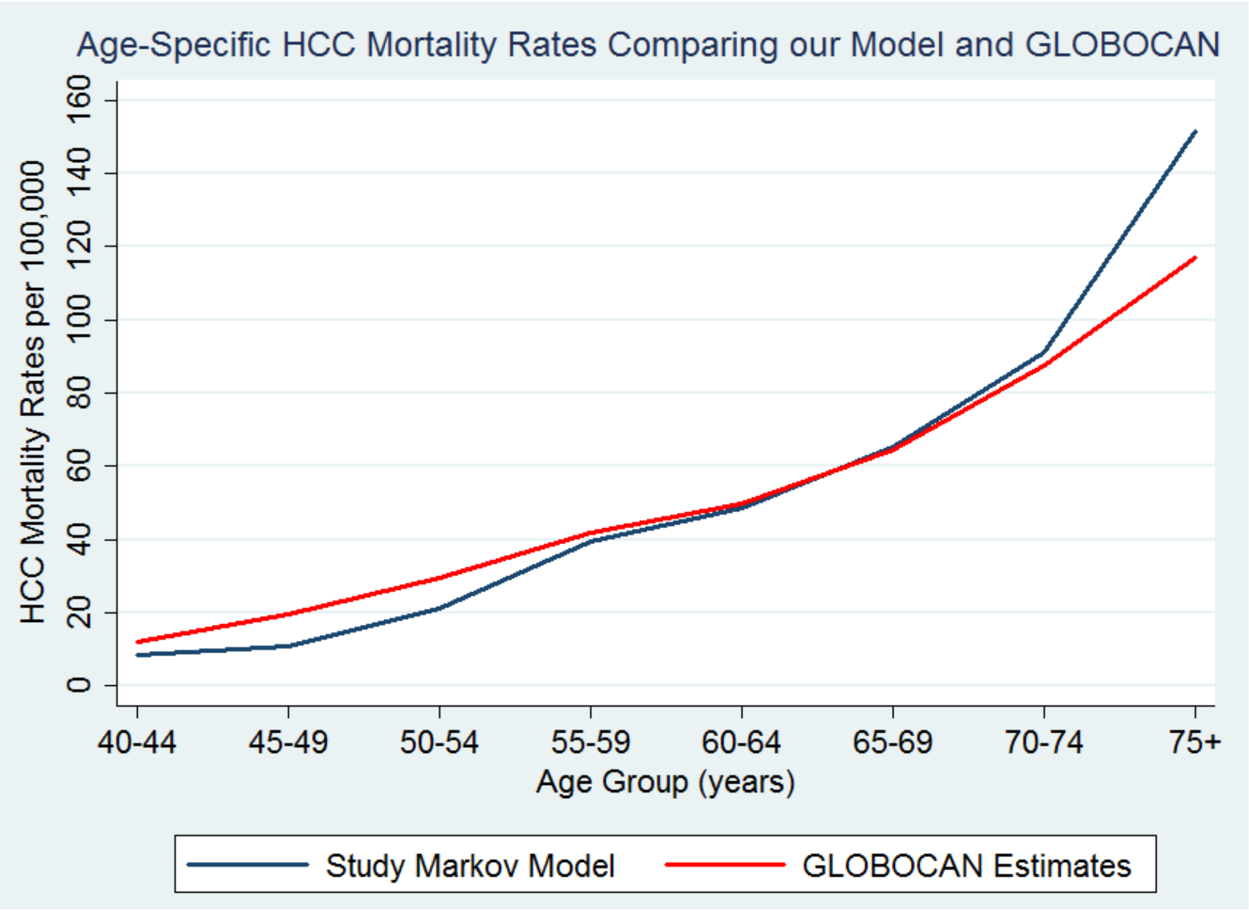

Appendix Figure 2: Cost-effectiveness acceptability curve for the probabilistic sensitivity analysis.

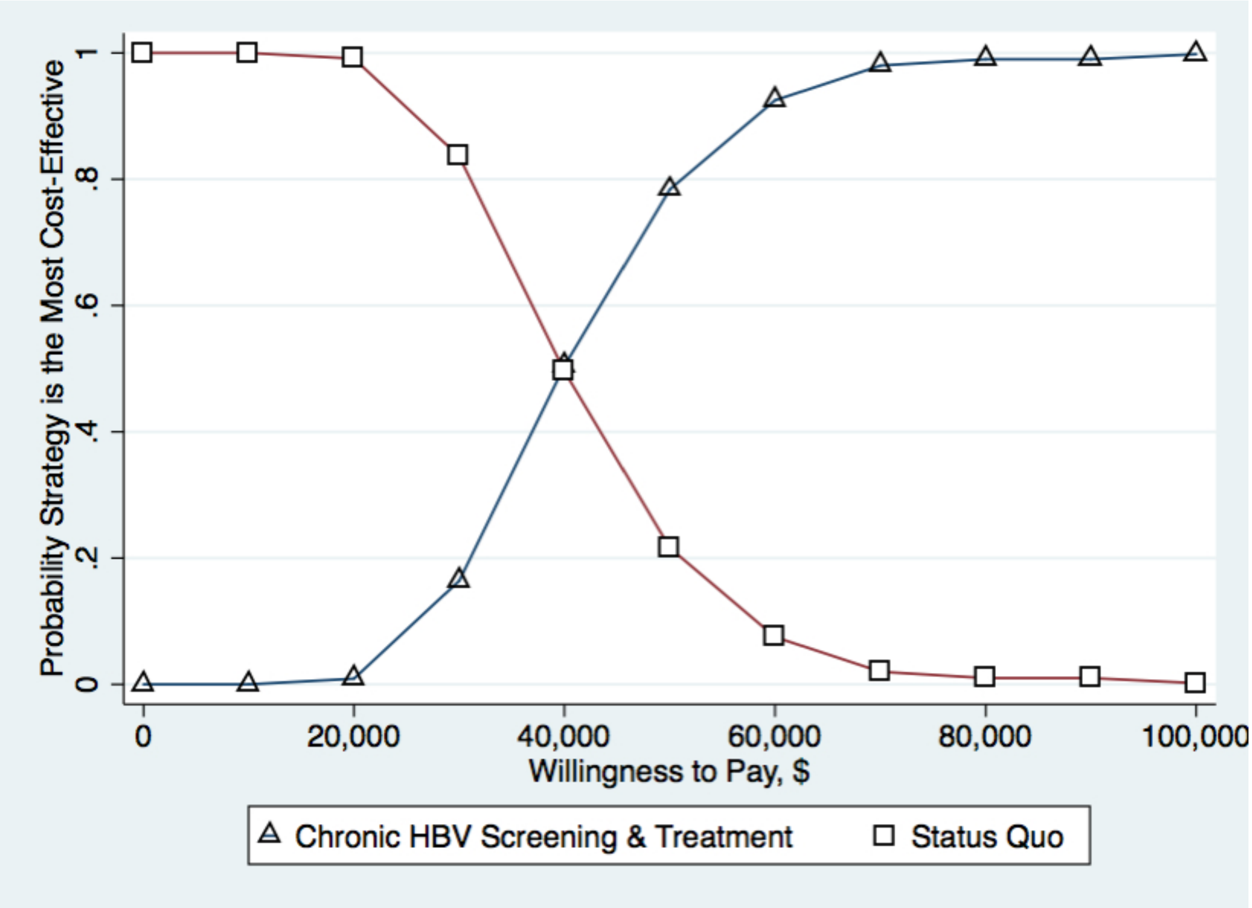

References

1. Rossi C, Shrier I, Marshall L, Cnossen S, Schwartzman K, et al. (2012) Seroprevalence of chronic hepatitis B virus infection and prior immunity in immigrants and refugees: A systematic review and meta-analysis. PLoS One 7: e44611.
2. Wong WW, Woo G, Heathcote EJ, Krahn M (2011) Cost effectiveness of screening immigrants for hepatitis B. Liver Int 31: 1179-1190.
3. Kao JH, Chen DS (2002) Global control of hepatitis B virus infection. Lancet Infect Dis 2: 395-403.
4. McMahon BJ (2010) Natural history of chronic hepatitis B. Clin Liver Dis 14: 381-396.
5. Hutton DW, Tan D, So SK, Brandeau ML (2007) Cost-effectiveness of screening and vaccinating Asian and Pacific Islander adults for hepatitis B. Ann Intern Med 147: 460-469.
6. Veldhuijzen IK, Toy M, Hahne SJ, De Wit GA, Schalm SW, et al. (2010) Screening and early treatment of migrants for chronic hepatitis B virus infection is cost-effective. Gastroenterology 138: 522-530.
7. Scheiblauer H, El-Nageh M, Diaz S, Nick S, Zeichhardt H, et al. (2010) Performance evaluation of 70 hepatitis B virus (HBV) surface antigen (HBsAg) assays from around the world by a geographically diverse panel with an array of HBV genotypes and HBsAg subtypes. Vox Sang 98: 403-414.
8. Huzly D, Schenk T, Jilg W, Neumann-Haefelin D (2008) Comparison of nine commercially available assays for quantification of antibody response to hepatitis B virus surface antigen. J Clin Microbiol 46: 1298-1306.
9. Mast EE, Weinbaum CM, Fiore AE, Alter MJ, Bell BP, et al. (2006) A comprehensive immunization strategy to eliminate transmission of hepatitis B virus infection in the United States: recommendations of the Advisory Committee on Immunization Practices (ACIP) Part II: immunization of adults. MMWR Recomm Rep 55: 1-33.
10. Dienstag JL (2008) Hepatitis B virus infection. N Engl J Med 359: 1486-1500.
11. Public Health Agency of Canada (2011) Brief report: Hepatitis B infection in Canada. Available: http://www.phac-aspc.gc.ca/id-mi/pdf/hepB-eng.pdf Accessed 2011 Mar 05.
12. Daniels D, Grytdal S, Wasley A (2009) Surveillance for acute viral hepatitis - United States, 2007. MMWR Surveill Summ 58: 1-27.
13. Ichai P, Samuel D (2008) Etiology and prognosis of fulminant hepatitis in adults. Liver Transp 14: S67-79.
14. Tillmann HL, Zachou K, Dalekos GN (2012) Management of severe acute to fulminant hepatitis B: to treat or not to treat or when to treat? Liver Int 32: 544-553.
15. Shepard CW, Simard EP, Finelli L, Fiore AE, Bell BP (2006) Hepatitis B virus infection: epidemiology and vaccination. Epidemiol Rev 28: 112-125.
16. Lok AS, McMahon BJ (2007) Chronic hepatitis B. Hepatology 45: 507-539.
17. Statistics Canada (2011) Table 102-0551 - Deaths and mortality rate, by selected grouped causes, age group and sex.
18. McMahon BJ, Holck P, Bulkow L, Snowball M (2001) Serologic and clinical outcomes of 1536 Alaska Natives chronically infected with hepatitis B virus. Ann Intern Med 135: 759-768.
19. Lin X, Robinson NJ, Thursz M, Rosenberg DM, Weild A, et al. (2005) Chronic hepatitis B virus infection in the Asia-Pacific region and Africa: review of disease progression. Journal of gastroenterology and hepatology 20: 833-843.

20. Chen CJ, Yang HI, Su J, Jen CL, You SL, et al. (2006) Risk of hepatocellular carcinoma across a biological gradient of serum hepatitis B virus DNA level. JAMA 295: 65-73.
21. Beasley RP (1988) Hepatitis B virus. The major etiology of hepatocellular carcinoma. Cancer 61: 1942-1956.
22. Iloeje UH, Yang HI, Su J, Jen CL, You SL, et al. (2006) Predicting cirrhosis risk based on the level of circulating hepatitis B viral load. Gastroenterology 130: 678-686.
23. Huo T, Wu JC, Hwang SJ, Lai CR, Lee PC, et al. (2000) Factors predictive of liver cirrhosis in patients with chronic hepatitis B: a multivariate analysis in a longitudinal study. Eur J Gastroenterol Hepat 12: 687-693.
24. Liaw YF, Tai DI, Chu CM, Lin DY, Sheen IS, et al. (1986) Early detection of hepatocellular carcinoma in patients with chronic type B hepatitis. A prospective study. Gastroenterology 90: 263-267.
25. Fattovich G, Pantalena M, Zagni I, Realdi G, Schalm SW, et al. (2002) Effect of hepatitis B and C virus infections on the natural history of compensated cirrhosis: a cohort study of 297 patients. Am J Gastroenterol 97: 2886-2895.
26. Chen DS (1994) Hepatitis B and C virus infections in hepatocellular carcinoma and their prevention. In: Nishioka K, editor. Viral Hepatitis and Liver Disease. Tokyo: Springer-Verlag. pp. 685-689.
27. Iloeje UH, Yang HI, Jen CL, Su J, Wang LY, et al. (2007) Risk and predictors of mortality associated with chronic hepatitis B infection. Clin Gastroenterol Hepatol 5: 921-931.
28. Hui AY, Chan HL, Leung NW, Hung LC, Chan FK, et al. (2002) Survival and prognostic indicators in patients with hepatitis B virus-related cirrhosis after onset of hepatic decompensation. J Clin Gastroenterol 34: 569-572.
29. Wong RJ, Corley DA (2009) Survival differences by race/ethnicity and treatment for localized hepatocellular carcinoma within the United States. Dig Dis Sci 54: 2031-2039.
30. Kanwal F, Gralnek IM, Martin P, Dulai GS, Farid M, et al. (2005) Treatment alternatives for chronic hepatitis B virus infection: a cost-effectiveness analysis. Ann Intern Med 142: 821-831.
31. Chen J, Sankaranarayanan R, Shen Z (1998) [Population-based cancer survival: an analysis of 16,922 cases]. Chinese Journal of Oncology 20: 202-206.
32. Sherman M, Shafran S, Burak K, Doucette K, Wong W, et al. (2007) Management of chronic hepatitis B: consensus guidelines. Can J Gastroenterol 21 (Suppl C): 5-24.
33. Scientific Registry of Transplant Recipients (2010) Annual Report Table 9.12b: Adjusted Patient Survival, Living Donor Liver Transplants - Survival at 3 Months, 1 Year, 5 Years, and 10 Years.
34. Koniaris LG, Levi DM, Pedroso FE, Franceschi D, Tzakis AG, et al. (2011) Is surgical resection superior to transplantation in the treatment of hepatocellular carcinoma? Ann Surg 254: 527-537.
35. Weinstein MC, Siegel JE, Gold MR, Kamlet MS, Russell LB (1996) Recommendations of the Panel on Cost-effectiveness in Health and Medicine. JAMA 276: 1253-1258.
36. Montreal Public Health Department (2011) Cost of Hepatitis B Vaccination. *Personal Correspondance*.
37. Jewish General Hosptial (2012) Cost of Serologic Testing. *Personal Correspondance*.
38. Regie de l'Assurance Maladie de Québec (2011) Manuel des medicins specialistes. Available:

http://www.ramq.gouv.qc.ca/sitecollectiondocuments/professionnels/manuels/150-facturation-specialistes/000_complet_acte_spec.pdf Accessed 2012 Jan 22.
39. British Columbia Ministry of Health (2012) Medical services commission payment schedule. Available: http://www.health.gov.bc.ca/msp/infoprac/physbilling/payschedule/index.html Accessed 2012 Feb 08.
40. Margolis HS, Coleman PJ, Brown RE, Mast EE, Sheingold SH, et al. (1995) Prevention of hepatitis B virus transmission by immunization. An economic analysis of current recommendations. JAMA 274: 1201-1208.
41. Arteaga-Rodriguez A, Carrasco-Garrido P, Lopez de Andres A, Santos J, Gil de Miguel A, et al. (2010) Trends of acute hepatitis B hospitalizations, comorbidities, fatality rate, and costs associated with the hospitalization in Spain (2001-2006). Eur J Gastroenterol Hepat 22: 961-966.
42. Gagnon YM, Levy AR, Iloeje UH, Briggs AH (2004) Treatment costs in Canada of health conditions resulting from chronic hepatitis B infection. J Clin Gastroenterol 38: S179-186.
43. Regie de l'Assurance Maladie de Québec (2012) Liste de medicaments. Available: http://www.ramq.gouv.qc.ca/fr/regie/publications-legales/pages/liste-medicaments.aspx Accessed 2012 Feb 14.
44. Sarasin FP, Giostra E, Mentha G, Hadengue A (1998) Partial hepatectomy or orthotopic liver transplantation for the treatment of resectable hepatocellular carcinoma? A cost-effectiveness perspective. Hepatology 28: 436-442.
45. Guerriere DN, Zagorski B, Fassbender K, Masucci L, Librach L, et al. (2010) Cost variations in ambulatory and home-based palliative care. Palliat Med 24: 523-532.
46. Levy AR, Kowdley KV, Iloeje U, Tafesse E, Mukherjee J, et al. (2008) The impact of chronic hepatitis B on quality of life: a multinational study of utilities from infected and uninfected persons. Value Health 11: 527-538.
47. International Agency for Research on Cancer (2011) Cancer Incidence in Five Continents Annual Dataset. Available: http://ci5.iarc.fr/CI5plus/ci5plus.htm Accessed 2012 Jul 05.
